# The effect of offering a third‐trimester routine ultrasound on pregnancy‐specific anxiety and mother‐to‐infant bonding in low‐risk women: A pragmatic cluster‐randomized controlled trial

**DOI:** 10.1111/birt.12573

**Published:** 2021-07-20

**Authors:** Myrte Westerneng, Ank de Jonge, Anneloes L. van Baar, Anke B. Witteveen, Petra Jellema, K. Marieke Paarlberg, Marlies Rijnders, Henriëtte E. van der Horst

**Affiliations:** ^1^ Midwifery Science AVAG Amsterdam Public Health Research Institute Amsterdam UMC Vrije Universiteit Amsterdam Amsterdam The Netherlands; ^2^ Child and Adolescent Studies Utrecht University Utrecht The Netherlands; ^3^ Department of Obstetrics and Gynaecology Gelre Hospitals Apeldoorn The Netherlands; ^4^ Department of Child Health TNO Leiden The Netherlands; ^5^ Department of General Practice and Elderly Care Medicine Amsterdam Public Health Research Institute Amsterdam UMC Vrije Universiteit Amsterdam Amsterdam The Netherlands

**Keywords:** cluster‐randomized trial, maternal bonding, pregnancy anxiety, third‐trimester routine ultrasound

## Abstract

**Background:**

Third‐trimester routine ultrasounds are increasingly offered to monitor fetal growth. In addition to limited evidence for its clinical effectiveness, little is known about its importance for pregnancy‐specific anxiety and mother‐to‐infant bonding.

**Methods:**

1275 low‐risk women participated in a Dutch nationwide pragmatic cluster‐randomized trial and answered questionnaires on pregnancy‐specific anxiety (PRAQ‐R) and prenatal mother‐to‐infant bonding (MAAS) before and after a third‐trimester routine ultrasound was offered to the intervention group. Linear mixed model regression analyses were performed to examine the effect of offering a third‐trimester routine ultrasound on pregnancy‐specific anxiety and mother‐to‐infant bonding. In addition, we examined whether the effect depended on maternal background characteristics and level of satisfaction with the ultrasound procedure.

**Results:**

We found no effect of offering a third‐trimester routine ultrasound on pregnancy‐specific anxiety and mother‐to‐infant bonding. However, interaction analyses showed that women with high levels of depressive symptoms at baseline and women who were very satisfied with the ultrasound procedure benefited somewhat more from offering a third‐trimester routine ultrasound in terms of mother‐to‐infant bonding compared with women with low or no depressive symptoms, or less satisfied women.

**Conclusions:**

The relationship between offering a third‐trimester routine ultrasound with pregnancy‐specific anxiety and mother‐to‐infant bonding is limited. A beneficial effect only applies to some subgroups of women. This implies that, in terms of psychological outcomes, there are no counterarguments to implementing a third‐trimester routine ultrasound. Strong evidence for offering all pregnant women a third‐trimester routine ultrasound for psychological reasons, however, is lacking.

## 1 INTRODUCTION

During the last three decades, ultrasounds have become an integral part of pregnancy‐related care in high‐resource countries.^1^, ^2^ In The Netherlands, women are currently offered at least two ultrasounds, which are covered by health care insurance: the dating scan at the beginning of pregnancy and the fetal anomaly scan at approximately 20 weeks of gestation. Recently, primary care midwives who take care of low‐risk women in The Netherlands increasingly offer third‐trimester routine ultrasounds to monitor fetal growth since the effectiveness of serial fundal height assessments has been found to be limited.^3^, ^4^, ^5^ Monitoring fetal growth is important, as intrauterine growth restriction is associated with a higher risk of perinatal mortality^6^ and morbidity.^7^, ^8^

However, firm evidence for the effectiveness of a third‐trimester routine ultrasound in reducing severe perinatal outcomes is lacking.^9^ The IUGR Risk Selection (IRIS) study, a large pragmatic cluster‐randomized controlled trial, was designed to evaluate the (cost‐) effectiveness of offering third‐trimester routine ultrasounds in reducing severe perinatal outcomes in low‐risk pregnant women.^10^ In this study, which is part of the larger IRIS study, we focused on one of the subgoals of the IRIS study and examined the experiences of low‐risk pregnant women with a third‐trimester routine ultrasound.

Ultrasounds offer women a chance to get information about the health of their child. In case of no abnormalities, this experience is believed to provide reassurance about the well‐being of the child and thereby reduce pregnancy‐specific anxiety levels.^11^, ^12^ In addition, getting a real‐time image of their child has been suggested to contribute to prenatal mother‐to‐infant bonding.^11^, ^13^ Both pregnancy‐specific anxiety and mother‐to‐infant bonding have received considerable attention over the years, since pregnancy‐specific anxiety and mother‐to‐infant bonding difficulties are associated with an increased likelihood of adverse neonatal outcomes^14^, ^15^ and poorer executive functioning and social‐emotional development of the child.^16^, ^17^

Evidence for a positive effect of routine ultrasounds on pregnancy‐specific anxiety and mother‐to‐infant bonding is limited.^18^ The role of ultrasounds in reducing maternal anxiety and increasing mother‐to‐infant bonding has only been suggested in qualitative studies^19^, ^20^ and in observational studies without a control group.^21^, ^22^, ^23^ The few conducted trials failed to find an effect.^24^, ^25^, ^26^ Furthermore, most studies have focused on ultrasounds in the first two trimesters and were performed in the 1980s and 90s when routine ultrasound technology was relatively new. This limits the generalizability of previous findings.

The aim of this study was to gain insight into the relationship of a third‐trimester routine ultrasound with pregnancy‐specific anxiety and mother‐to‐infant bonding. This insight is needed to make a balanced decision about introducing a third‐trimester ultrasound as routine. Our primary research question is: What is the effect of offering a third‐trimester routine ultrasound on pregnancy‐specific anxiety and prenatal mother‐to‐infant bonding in current antenatal care practice? Our second research question examined whether certain subgroups of women benefit more from a routine ultrasound than others. Specifically, we assessed whether baseline levels of pregnancy‐specific anxiety and mother‐to‐infant bonding (*research question 2a*), background characteristics (*research question 2b*), and the level of satisfaction with the ultrasound procedure (*research question 2c*) are potential moderators.

## 2 METHODS

### 2.1 Participants and procedure

The IRIS study is a cluster‐randomized controlled trial carried out among 60 primary care midwifery practices across The Netherlands. Between February 2015 and February 2016, all singleton low‐risk pregnant women who were at least 16 years old and in midwife‐led care at the time of inclusion (20‐24 weeks of gestation) were informed about the IRIS study by their midwife. In addition, a leaflet was provided. Women in the intervention group were informed that they would be offered two additional ultrasounds in their third trimester to monitor fetal growth—one between 28 and 30 weeks of gestation and one between 34 and 36 weeks of gestation. About one third of sonographers taking part in the study were primary care midwives who performed the ultrasound scans in their own practice. The others were working in ultrasound centers and hospitals and were not involved in the (primary) care offered to the woman. Details of the study design are described elsewhere.^10^

Between May and December 2015, a random subsample of participants enrolled in the IRIS study was invited to fill out questionnaires on maternal and neonatal physical health, maternal mental health (including pregnancy‐specific anxiety), mother‐to‐infant bonding, and use of and experiences with health care. As prespecified in the IRIS study design, at least 450 women in the control group and 450 women in the intervention group were to be included to be able to perform cost‐effectiveness analyses by applying Bayesian techniques in combination with Monte Carlo simulation.^10^ For our research questions, this sample size allowed us to include a minimum of 45 and a maximum of 60 predictor variables, based on a standard of 15‐20 cases per predictor variable.^27^, ^28^ After informed consent was obtained, women received two prenatal questionnaires, which were sent before (between 20 and 27 weeks of gestation, T1) and after (around 32 weeks of gestation, T2) the first third‐trimester routine ultrasound was offered to the intervention group. Consequently, our study only focused on the psychological effect of the *first* third‐trimester routine ultrasound. If women did not fill out the questionnaires, two reminders were sent by e‐mail. Only participants who filled out at least one of our main outcome measures (pregnancy‐specific anxiety or mother‐to‐infant bonding) at T1 and T2 were included in the current study.

### 2.2 Measures

#### 2.2.1 Psychological variables

Pregnancy‐specific anxiety (T1 and T2) was measured with the 10‐item adjusted version of the Pregnancy‐Related Anxiety Questionnaire (PRAQ‐R2)^29^ consisting of three subscales: “fear of giving birth” (3 items), “fear of bearing a handicapped child” (4 items), and “concerns about one's appearance” (3 items). Items are rated on a 5‐point Likert scale, ranging from definitely not true to definitely true. Total scores range between 10 and 50, with higher scores indicating higher levels of pregnancy‐specific anxiety. Sufficient reliability and validity has been shown for both primiparous and multiparous women.^29^

Prenatal mother‐to‐infant bonding (T1 and T2) was measured with the 19‐item Maternal Antenatal Attachment Scale (MAAS)^30^ consisting of two subscales: “intensity of preoccupation” with the fetus (8 items) and “quality” of the mother's feelings toward the fetus (11 items). The total score ranges between 19 and 95. Higher scores indicate higher levels of prenatal bonding. The MAAS has been found to be a reliable and valid measure of antenatal attachment.^30^, ^31^

Depressive symptoms (T1) were measured with the shortened version of the Edinburgh Depression Scale (EDS).^32^ Ten items are rated on 4‐point Likert scales. The total score ranges between 0 and 30, and higher scores indicate more symptoms of depression. Scores above a cutoff level of 10 indicate severe depressive complaints in the second and third trimester.^31^ The scale has been validated for both antenatal and postnatal usage.^33^, ^34^, ^35^, ^36^

General anxiety (T1) was measured with the six‐item short form of the state scale of the Spielberger State‐Trait Anxiety Inventory (STAI).^37^ Items are rated on 4‐point scales, resulting in a sum score ranging from 6 to 24. Higher scores represent higher general anxiety levels. This measurement is a valid instrument for both pregnant and nonpregnant populations.^38^

#### 2.2.2 Ultrasound exposure

In the questionnaire at T2, women were asked whether they had received any ultrasounds since the completion of questionnaire T1, and if so, how many of these were initiated by their midwife (either routine or clinically indicated) and how many by means of their own request (keepsake). Based on this information and the timing of the ultrasounds registered in the ultrasonography data, the number of ultrasounds between T1 and T2 was extracted. Women in the intervention group who received at least one midwife‐initiated ultrasound between T1 and T2, and who received an ultrasound within the period prespecified for the first routine ultrasound, were marked as having received a third‐trimester routine ultrasound.

#### 2.2.3 Fetal growth

Based on the growth measurements provided in the ultrasound data, we determined whether there was suspicion of growth restriction, macrosomia, or decreased amniotic fluid—the three most likely adverse outcomes of a third‐trimester routine ultrasound. In accordance with the IRIS study protocol, we defined suspicion of growth restriction as an abdominal circumference below the 10th percentile, suspicion of macrosomia as an abdominal circumference above the 90th percentile, and decreased amniotic fluid as the deepest pocket below two centimeters.

#### 2.2.4 Satisfaction with the ultrasound procedure

We developed a questionnaire of 11 items to measure how satisfied women were with the ultrasound procedure. The questionnaire consisted of items measuring how women generally experienced information received before a midwife‐initiated ultrasound (2 items), the experience of the ultrasound itself (7 items), and the information received about the results of the ultrasound afterward (2 items). Items were rated on 5‐point Likert scales (fully disagree to fully agree). Sum scores could range between 11 and 55, with higher scores indicating a higher degree of satisfaction with the ultrasound procedure. Cronbach's alpha was found to be good (α = 0.91). The full list of items is presented in the Appendix 1.

#### 2.2.5 Sociodemographic variables

At the time of enrollment in the IRIS study, participants reported their date of birth and expected date of delivery, and other sociodemographic background information.

### 2.3 Statistical analyses

We performed descriptive statistics (eg, means, frequencies) to describe the study sample and t tests and chi‐square tests to explore differences in background characteristics between the control group and the intervention group. To answer *research question 1*, whether offering a third‐trimester routine ultrasound affects pregnancy‐specific anxiety and mother‐to‐infant bonding in current maternity care, we performed linear mixed model analyses. In the first model, we only entered condition (intervention vs control group) as a fixed effect. In the subsequent models, we consecutively added the following: (a) midwifery practice to account for the clustered nature of the data; (b) baseline (T1) levels of our outcomes of interest (pregnancy‐specific anxiety and mother‐to‐infant bonding); and (c) baseline levels of background characteristics associated with pregnancy‐specific anxiety^39^, ^40^, ^41^ and mother‐to‐infant bonding.^42^ Midwifery practice was added as a random effect; all other variables were added as fixed effects. Since our focus was on real‐world maternity practice, we followed a pragmatic design in our main analyses. As a per‐protocol analysis, we repeated these analyses but excluded women from the control group who received ultrasounds between T1 and T2. From the intervention group, we excluded women who received no ultrasounds and women who received nonroutine ultrasounds. Any effect of a third‐trimester routine ultrasound that might be blurred by the effects of ultrasounds received other than a third‐trimester routine ultrasound (clinically indicated or keepsake) should become visible in this analysis. In addition, as a sensitivity analysis, we further excluded women in the intervention group with suspicion of fetal growth restriction, macrosomia, or decreased amniotic fluid based on a third‐trimester routine ultrasound and compared them with women in the control group who did not receive any ultrasounds. In this analysis, the effects of receiving a third‐trimester routine ultrasound were least likely to be confounded by clinical factors. For all analyses, in addition to the sum scores of pregnancy‐specific anxiety and mother‐to‐infant bonding, we also analyzed the PRAQ‐R2 subscale “fear of bearing a handicapped child.” We hypothesized that a third‐trimester routine ultrasound may affect child‐related concerns, but not concerns about one's appearance or fear of giving birth.

Next, we repeated the mixed model analyses to assess whether the following two sets of variables measured at baseline (T1) moderated the effect: pregnancy‐specific anxiety and mother‐to‐infant bonding (*research question 2a*) and background characteristics associated with pregnancy‐specific anxiety and mother‐to‐infant bonding (*research question 2b*). Based on Fournier and colleagues,^43^ we first entered main effects and interaction terms to our mixed model for each set of variables. For research question 2b, in which multiple interaction effects were tested, interaction terms with *P* values below 0.20, 0.10, and 0.05 were retained in a stepwise fashion. Main effects were retained as long as the interaction terms were retained. We used the Web program ModGraph to obtain simple slopes of the associations for the control and the intervention group. To analyze whether satisfaction with the ultrasound procedure is a moderator (*research question 2c*), we repeated the mixed model analyses, only this time condition was divided into three groups: a control group, an intervention group who scored above the 50th percentile (score ≥44) on the ultrasound satisfaction scale, and an intervention group who scored below the 50th percentile on the ultrasound satisfaction scale (score <44).

## 3 RESULTS

### 3.1 Background characteristics of the study sample at baseline (T1)

**FIGURE 1:**
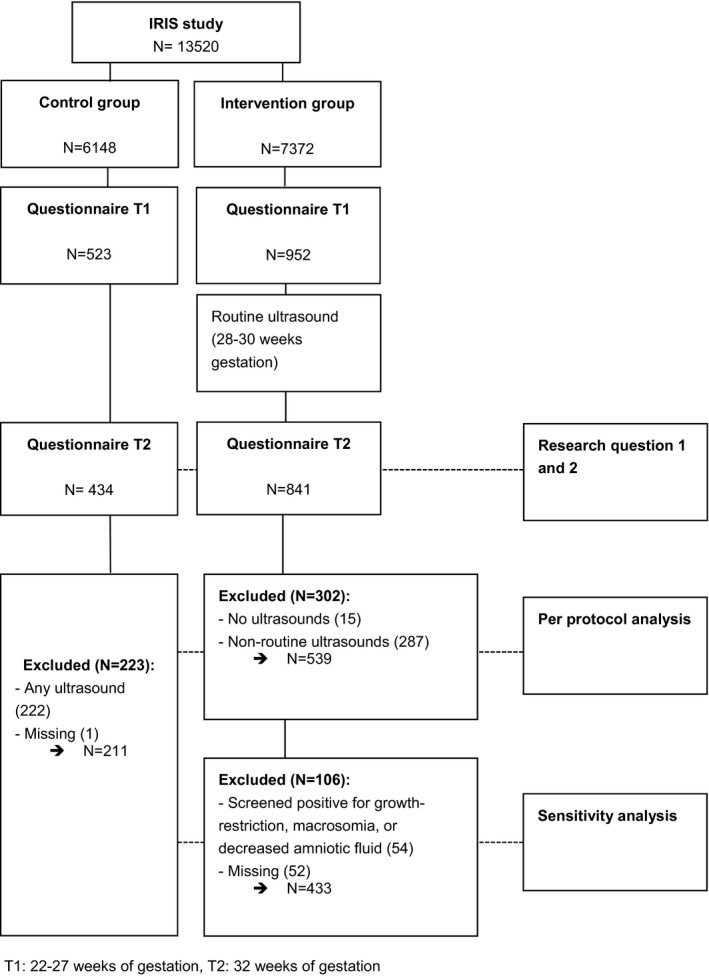
Flow chart of the study sample

A subsample of 1475 women (N control group = 523 and N intervention group = 952) enrolled in the IRIS study and answered the first questionnaire (T1). From this group of women, 83% of the control group (N = 434) and 88.3% (N = 841) of the intervention group completed T2, leaving a total of 1275 respondents for further analyses (Figure 1). Questionnaire T1 was completed at a mean of 24.1 (sd = 1.96) weeks of gestation; questionnaire T2 was filled out at a mean of 32.1 (sd = 0.72) weeks of gestation. Women in the control group were similar to women in the intervention group in background characteristics and baseline levels of psychological symptoms, except for ethnicity (Table 1). In the intervention group, more women were of non‐Dutch ethnicity (19.1%) than in the control group (13.4%), χ^2^ (1) = 6.64, *P* = 0.01. Our total sample was representative of the Dutch population in terms of maternal age and parity.^44^ Women with high education levels and women of Dutch ethnicity were overrepresented.^45^

**TABLE 1:** Baseline (T1) characteristics of the study sample (N = 1275)^a^

		Control group (N = 434)	Intervention group (N = 841)	*P*
Age (years)	Mean (sd)	31.65 (3.84)	31.58 (4.24)	0.78
	<25	19 (4.4)	45 (5.4)	0.71
	25‐35	321 (74.1)	609 (72.5)	
	>35	93 (21.5)	186 (22.1)	
	Missing	1	1	
Parity	Primiparous	209 (48.5)	409 (48.9)	0.88
	Multiparous	222 (51.5)	427 (51.1)	
	Missing	3	5	
Ethnicity	Dutch	375 (86.6)	680 (80.9)	0.01
	Non‐Dutch	58 (13.4)	161 (19.1)	
	Missing	1	0	
Educational level	Low	30 (6.9)	55 (6.6)	0.15
	Moderate	130 (30.1)	395 (35.6)	
	High	272 (63.0)	479 (57.8)	
	Missing	2	12	
Relationship with father unborn child	Yes	426 (98.6)	810 (97.2)	0.12
	No	6 (1.4)	23 (2.8)	
	Missing	2	8	
Pregnancy‐specific anxiety (PRAQ‐R2)	Mean (sd)	19.94 (5.83)	20.14 (6.21)	0.58
	Missing	10	15	
Mother‐to‐infant bonding (MAAS)	Mean (sd)	75.70 (5.81)	76.21 (6.13)	0.16
	Missing	12	26	
General anxiety (STAI)	Mean (sd)	9.75 (2.84)	10.05 (2.90)	0.08
	Missing	5	12	
Depressive symptoms (EDS)	Mean (sd)	5.48 (4.26)	5.82 (4.02)	0.16
	Missing	7	21	

^a^ Results are presented as N (%), unless stated otherwise

### 3.2 Ultrasound exposure

In the intervention group, 826 women (98.2%) received an ultrasound between T1 and T2, whereas 222 women (51.3%) in the control group received at least one ultrasound during this period. Among women in the intervention group who received one or more ultrasounds between T1 and T2, 539 women (65.3%) received a routine ultrasound only.

### 3.3 Fetal growth

For 487 (90.4%) of 539 women in the intervention group who received a routine ultrasound only, ultrasonography data were available. We found that in 54 (11.1%) women, fetal growth restriction, macrosomia, or decreased amniotic fluid were suspected.

### 3.4 The effect of offering a third‐trimester routine ultrasound on pregnancy‐specific anxiety and mother‐to‐infant bonding (research question 1)

Both the unadjusted model and the final adjusted model examining the effect of offering a third‐trimester routine ultrasound on sum scores of the PRAQ‐R2 and MAAS at T2 are presented in Table 2. No differences between the control and the intervention group were found. In addition, no differences were found for “fear of bearing a handicapped child.”

**TABLE 2:** Effect of offering a third‐trimester routine ultrasound on pregnancy‐specific anxiety and mother‐to‐infant bonding obtained from linear mixed model analysis

	Control group (N = 434)	Intervention group (N = 841)	B (95% CI)	*P*‐value
	Mean (sd)	Mean (sd)		
Pregnancy‐specific anxiety sum score (PRAQ‐R2 T2)				
Unadjusted model	19.74 (5.62)	19.65 (6.05)	−0.09 (−0.78 to 0.60)	0.81
Final model			−0.31 (−0.74 to 0.11)	0.15
Mother‐to‐infant bonding sum score (MAAS T2)				
Unadjusted model	74.42 (5.76)	75.04 (5.67)	0.65 (−0.07 to 1.37)	0.08
Final model			0.37 (−0.18 to 0.92)	0.18

Note

In the unadjusted model, the crude associations are presented. In the final model, we adjusted for midwifery practice, baseline levels of our outcomes of interest, baseline levels of general anxiety, and depressive symptoms for both our outcomes of interest and parity and ethnicity when examining pregnancy‐specific anxiety. Sample sizes slightly differ per analysis because of missings on outcomes or confounding variables.

Table 3 presents the results of our per‐protocol analysis in which we excluded 223 women from the control group who received any ultrasound, and 287 women from the intervention group who received nonroutine ultrasounds; 15 women in the intervention group who received no ultrasounds were excluded. Women in the intervention group who only received a routine ultrasound between T1 and T2 (n = 539) scored lower on the PRAQ‐R2 sum score at T2 than women in the control group who received no ultrasounds within this period (B (95% CI) = −0.61 (−1.18 to −0.04), *P* =.04) in the final model. No differences were found for “fear of bearing a handicapped child” or for the MAAS sum score. The sensitivity analysis, in which we additionally excluded 54 women from the intervention group with suspicion of fetal growth restriction, macrosomia, or decreased amniotic fluid based on a third‐trimester routine ultrasound, yielded similar results (results not shown).

**TABLE 3:** Effect of a third‐trimester routine ultrasound on pregnancy‐specific anxiety and mother‐to‐infant bonding: per‐protocol analysis

	Control group (N = 211)	Intervention group (N = 539)	B (95% CI)	*P*‐value
	Mean (sd)	Mean (sd)		
Pregnancy‐specific anxiety sum score (PRAQ‐R2 T2)				
Unadjusted model	20.13 (5.60)	19.33 (5.83)	−0.80 (−1.73 to 0.13)	0.09
Final model			−0.61 (−1.18 to −0.04)	0.04
Mother‐to‐infant bonding sum score (MAAS T2)				
Unadjusted model	77.52 (5.86)	78.23 (5.86)	0.71 (−0.24 to 1.65)	0.14
Final model			0.38 (−0.36 to 1.11)	0.31

Note

In the unadjusted model, the crude associations are presented. In the final model, we adjusted for midwifery practice, baseline levels of our outcomes of interest, baseline levels of general anxiety and depressive symptoms for both our outcomes of interest and parity and ethnicity when examining pregnancy‐specific anxiety. Sample sizes slightly differ per analysis because of missings on outcomes or confounding variables.

### 3.5 Moderating factors

#### 3.5.1 Baseline (T1) levels of pregnancy‐specific anxiety and mother‐to‐infant bonding (research question 2a)

**FIGURE 2:**
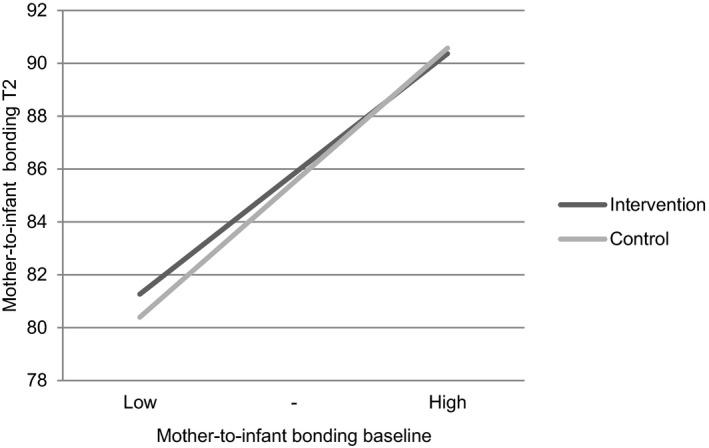
Interaction of baseline level of mother‐to‐infant bonding with the intervention on mother‐to‐infant bonding at T2

No significant interaction was found for baseline levels of the PRAQ‐R2 sum score and offering a third‐trimester routine ultrasound in our final model, indicating no differences in the effect of offering a third‐trimester routine ultrasound on pregnancy‐specific anxiety between women with different baseline levels of pregnancy‐specific anxiety. In addition, no interaction effect was found for the subscale “fear of bearing a handicapped child.” For the MAAS baseline sum score, a significant interaction with the intervention was found (B (95% CI) = −0.09 (−0.17 to −0.01), *P* = 0.03). Women with lower mother‐to‐infant bonding levels at baseline showed higher levels of mother‐to‐infant bonding at T2 in the intervention group compared with the control group (Figure 2).

#### 3.5.2 Background characteristics (research question 2b)

**FIGURE 3:**
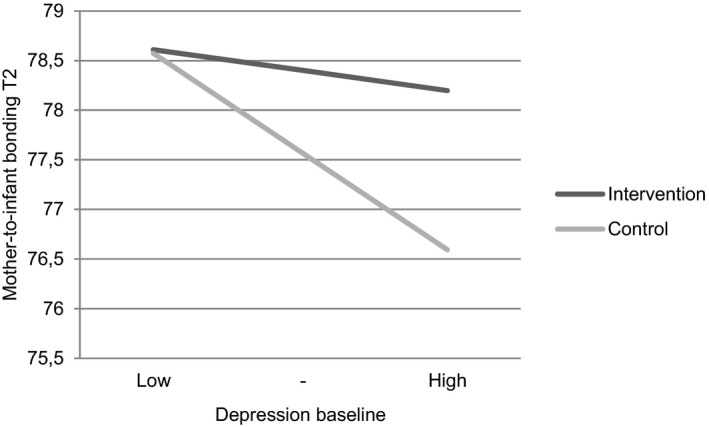
Interaction of baseline level of depressive symptoms with the intervention on mother‐to‐infant bonding at T2

No moderating effect was found for general anxiety, depressive symptoms, parity, and ethnicity when examining the PRAQ‐R2 sum score and “fear of bearing a handicapped child” subscale. However, women with higher baseline levels of depressive symptoms had higher mother‐to‐infant bonding levels at T2 in the intervention group than in the control group (B (95% CI) = 0.19 (0.02 to 0.36), *P* = 0.03) (Figure 3).

#### 3.5.3 Satisfaction with the ultrasound procedure (research question 2c)

Data on the moderating effect of satisfaction with the ultrasound procedure were available for 671 women in the intervention group who received a third‐trimester routine ultrasound. No significant differences were found for the PRAQ‐R2 sum score nor “fear of bearing a handicapped child.” The women with higher satisfaction scores in the intervention group scored higher on the MAAS at T2 than the control group (B (95% CI) = 0.82 (0.22 to 1.42), *P* = <0.01), whereas there was no significant difference between the women in the intervention group and the control group with lower satisfaction scores.

## 4 DISCUSSION

Our pragmatic trial shows that offering a third‐trimester routine ultrasound to low‐risk women in current Dutch antenatal care is not associated with pregnancy‐specific anxiety levels or mother‐to‐infant bonding levels. After excluding the substantial group of women who received nonroutine ultrasounds (clinically indicated or keepsake) from both the intervention and control group, we found that women who received a third‐trimester routine ultrasound scored somewhat lower on pregnancy‐specific anxiety than women who received no ultrasounds. In addition, we found that women with lower mother‐to‐infant bonding levels at baseline showed higher levels of mother‐to‐infant bonding at T2 in the intervention group compared with the control group. These differences, although statistically significant, were small, and therefore, the clinical relevance of these findings is questionable. A somewhat stronger effect concerned the protective effect of offering a third‐trimester routine ultrasound on mother‐to‐infant bonding for women with higher levels of depressive symptoms at baseline. In addition, women who were very satisfied with the ultrasound procedure seemed to benefit to some extent from being offered a third‐trimester routine ultrasound in terms of mother‐to‐infant bonding. This was not the case for women who were less satisfied.

Our results show that although offering a third‐trimester routine ultrasound does not have a psychological benefit for all pregnant women, it might be beneficial in terms of mother‐to‐infant bonding for women with higher levels of depressive symptoms. Depressive symptoms have been found to be negatively associated with mother‐to‐infant bonding.^42^, ^46^, ^47^ Possibly, getting a glimpse of the baby might be helpful for women who feel less connected to their baby because of depressive symptoms.

In addition, women who were very satisfied with their ultrasounds benefited slightly from being offered a third‐trimester routine ultrasound in terms of mother‐to‐infant bonding. This indicates that aspects of the ultrasounds procedure such as receiving clear and sufficient information and being able to ask questions might matter for psychological outcomes. As suggested by Whynes et al,^48^ the psychological impact of an ultrasound scan is not solely a result of the ultrasound scan itself, but largely depends on how women perceive the procedure. This perception is, in turn, affected partly by their satisfaction about the quality of the interaction with health care professionals.^49^, ^50^ In line with Nabhan and Faris,^26^ however, we did not find that satisfaction with the ultrasound procedure moderated the effect of an ultrasound on anxiety levels.

Our results align with a qualitative study, which showed that women do not feel that third‐trimester routine ultrasounds reduce pregnancy‐specific anxiety or improve the bond with their baby. Interestingly, women did seem to appreciate a third‐trimester routine ultrasound, which might arise from getting used to routine ultrasounds throughout pregnancy.^51^

We examined the psychological effects of offering a third‐trimester routine ultrasound in low‐risk pregnant women in a nationwide pragmatic trial. Next to the strengths of our study, such as a large and varied sample, some limitations should be mentioned. First, there is some discussion on whether the PRAQ‐R covers all facets of pregnancy‐specific anxiety.^52^ We encourage future studies to focus on a wider range of facets, including feelings of being confident or in control. Second, given the timing of the questionnaires, we were not able to include the effect of the second third‐trimester routine ultrasound offered in the IRIS study. It is possible that receiving two ultrasounds would have had more impact than one, especially for the subgroups who seem to benefit from a third‐trimester routine ultrasound.

In sum, our study shows that in terms of psychological outcomes, there are no counterarguments to implementing a third‐trimester routine ultrasound. At the same time, firm evidence for offering all pregnant women a third‐trimester routine ultrasound for psychological reasons is lacking. Balancing these results with the ineffectiveness of third‐trimester routine ultrasounds to improve birth outcomes, implementation of third‐trimester routine ultrasound is currently not warranted.

## Data Availability

The data that support the findings of this study are available from the corresponding author upon reasonable request.

## APPENDIX 1 Questionnaire to assess experiences with the third‐trimester routine ultrasound procedure

1. Prior to the ultrasound procedure, I received sufficient information about the ultrasound.
2. Prior to the ultrasound procedure, I received clear information about the ultrasound.
3. During the ultrasound procedure, the health care provider took enough time for me.
4. During the ultrasound procedure, I was sufficiently able to look at the screen myself.
5. During the ultrasound procedure, I could recognize (body parts of) the baby.
6. During the ultrasound procedure, the health care provider sufficiently explained what was visible on the screen.
7. During the ultrasound procedure, the health care provider clearly explained what was visible on the screen.
8. During the ultrasound procedure, I had the chance to ask questions to the health care provider.
9. During the ultrasound procedure, I had the chance to see how the baby reacted to my behavior (eg, touching the belly, talking).
10. After the ultrasound procedure, I got sufficient information about the ultrasound result.
11. After the ultrasound procedure, I got clear information about the ultrasound result.
